# Abortion Rights: Perspectives of Academic Scientists in the United States

**DOI:** 10.1089/whr.2024.0041

**Published:** 2024-09-04

**Authors:** Ashlee Frandell, Shaika Islam, Tipeng Chen, Mattia Caldarulo, Timothy P. Johnson, Lesley Michalegko, Yidan Zhang, Eric Welch

**Affiliations:** ^1^ University of Nevada Las Vegas, Las Vegas, Nevada, USA.; ^2^ Arizona State University, Phoenix, Arizona, USA.; ^3^ Rochester Institute of Technology, Rochester, New York, USA.; ^4^ University of Illinois Chicago, Chicago, Illinois, USA.

**Keywords:** abortion rights, Dobbs v Jackson, academic scientist opinions

## Abstract

In 2022, the US Supreme Court decision in *Dobbs v. Jackson Women’s Health Organization* to overturn federal law safeguarding abortion rights led to considerable national debate on abortion and reproductive rights. We report the findings of a survey of academic scientists’ perspectives regarding abortion rights, state policies, and the impact of the 2022 Supreme Court decision in *Dobbs v. Jackson*. Furthermore, we look at how academic scientists’ institutions acted to address the *Dobbs* decision. Using a 2023 cross-sectional survey, we address the following research questions: (i) What are scientists’ views of abortion rights? (ii) How have scientists responded to the 2022 Supreme Court decision in *Dobbs v. Jackson Women’s Health Organization*? and (iii) How are their views different from that of the general public with regard to *Dobbs v. Jackson* and abortion rights in general? Findings show that abortion was a key factor influencing scientists’ voting decisions. We also highlight substantial differences between scientists’ perspectives and those of the general population and reveal gender differences of opinions within the scientific community. We conclude by presenting the actions implemented by universities and scholars in response to the *Dobbs* decision and discuss the implications our results have for both policy and practice.

## Introduction

The US Supreme Court decision in *Roe v. Wade* in 1973 gave individuals federally protected rights to an abortion up to a certain point in their pregnancy.^1^ In June 2022, the Supreme Court overturned this ruling, leaving the legal battle over abortion to the states. Given the ideological and scientific nature of debates regarding abortion, scientists are an important constituency, both as experts and as citizens.^2,3^ As a result, and as the nation begins to debate these issues at the state level, it is critical to understand where the science community stands in terms of abortion rights. Therefore, we ask: What are academic scientists’ views of abortion rights? How have academic scientists responded to the 2022 Supreme Court decision *Dobbs v. Jackson Women’s Health Organization*? How do academic scientists’ views differ from those of the general public with regard to these issues?

We answer these questions using data from a 2023 survey of a nationally representative sample of biologists, geographers, public health scholars, and civil and environmental engineers on the faculty of Research One (R1) Carnegie-classified universities. The survey asks academic scientists about their perspectives on abortion rights and the implications of *Dobbs v. Jackson* for universities and the general population. Results indicate that abortion was a key indicating factor influencing scientists’ voting decisions. They also reveal gender differences of opinions within the scientific community.

Our study contributes to the extant literature on the social and ethical dimensions of the Supreme Court *Dobbs* decision by offering a comprehensive nationwide perspective on university researchers’ views related to the abortion debate. Moreover, we focus on academic scientists’ perspectives, actions, and the measures implemented by universities following the overturning of *Roe v. Wade*. In the next section, we overview the *Dobbs* decision and outline key surveys of the general public and other research on abortion rights in the United States. We then report data, methods, and findings from our nationally representative survey of academic scientists. We conclude with a discussion of the implications for research and policy.

## Literature Review

While public debates on abortion rights in the United States go back to the 19th century when the first antiabortion movement emerged,^4,5^ it was not until 1973 that the US Supreme Court ruled in favor of abortion rights (*Roe v. Wade* decision^a^). In June 2022, the Supreme Court overturned this landmark decision in *Dobbs v. Jackson,*^b^ reversing 50 years of federal law safeguarding abortion rights. The decision raised a national debate on abortion, human rights, and reproductive health that influenced the 2022 midterm election.^6–8^

According to Jozkowski et al. (2023),^9^ individuals involved in abortion activities include those seeking an abortion, abortion service providers, individuals responsible for the pregnancy, those offering any information or support to the person seeking an abortion, in the case of underage pregnant women—the parents; and lastly those who are doing research on abortion and supporting it.^9,10^ Research has examined the perceptions of the general public around abortion activities and reproductive rights, group differences in opinion (*e.g.,* men v. women) about legalizing abortion, and how these beliefs shaped voting decisions during the 2022 midterm elections.^4,11,12^ For example, Crawford and colleagues (2023),^13^ PEW Research (2022),^11^ and Kaiser Family Foundation (KFF, 2022)^14^ examined public attitudes about abortion rights and views on the *Dobbs* decision, particularly whether abortion should be legal or not. These studies consistently show public support for the legalization of abortion in all circumstances or with certain restrictions.^11,13^ Furthermore, studies have found that religion, education, and income/employment significantly shape the attitudes people have toward abortion.^15^ Meanwhile, Jozkowski et al. (2023)^16^ revealed that in a sample of the general public, approximately 36% believe abortion should never be banned, in contrast to the 20.5% who feel that abortion should always be banned. These responses were further broken down by circumstances, with almost half the respondents indicating that abortion should be made legal in cases where the pregnant person’s life is at risk or in instances of rape.^16^

Although these studies shed light on general US public opinion regarding abortion rights, they overlook scientists as an important subgroup to consider. Research has shown that the public generally agrees that scientists play an important role in these national policy discussions. One study, for example, found that about 80% of Americans believe that abortion policy should be left to biologists due to the nature of the subject.^2^ As a result, understanding the opinions of academic scientists and other experts regarding abortion may be crucial to ascertaining the potential future consequences of the Supreme Court’s decision to overrule *Roe v. Wade*.^17^

In addition, due to the nature of this topic, it is unclear whether there are differences of opinions by gender among academic scientists similar to those observed among the general public. Research shows that women coming from historically marginalized and underserved groups are least informed and knowledgeable about reproductive biology and more influenced by religious and ideological beliefs.^18^ Yet, they are also those who will most likely be most affected by the Supreme Court decision.^19^ Moreover, men are usually less likely to express support for legal abortion.^11^ Exploring the opinions among STEM faculty, where women are often underrepresented and face challenges in having their voices heard,^20,21^ should fill in this gap of understanding gender disparities within the scientific community regarding these issues and how reflective they are of those within the broader population.

While in the aftermath of the recent Supreme Court decision editorials have been published in several scientific journals to address this topic, these contributions may provide a representation of the academic world that is less nuanced than what actually exists as editorial boards may not be representative of the academic population at large.^22^ A more in-depth analysis of academic scientists’ perspectives would provide a better representation of scientists’ opinions around the debate.

Last, although some universities have responded publicly to the Supreme Court decision by condemning the ruling, a few welcomed it, and some opted for neutral statements or refrained from commenting.^23^ Overall, less is known about the position academia has taken following the overruling of *Roe v. Wade*, with evidence being mostly anecdotal. Such an analysis may provide insights into the actual involvement of academic scientists and universities in the policy and political landscape and indicate how STEM faculty beliefs and ideas regarding abortion rights are translated into actions.

Our study addresses important gaps by asking academic scientists their opinions on this topic following *Dobbs v. Jackson*. Results presented below provide some initial evidence regarding their views on abortion rights. Moreover, we investigate researchers’ and universities’ responses to the Supreme Court decision. Results have important implications as they illustrate the difference of opinions existing within and between academics as well as universities’ institutional involvement in the policy domain.

## Survey Analysis

### Sampling and survey administration

The cross-sectional survey was administered to a sample of SciOPS panel^c^ members using a two-stage sampling design. First, in the spring of 2022, we recruited 986 academic scientists to join our SciOPS panel from a sample frame that represents PhD-level faculties at Carnegie-designated Research Extensive and Intensive (R1) universities in the United States with faculty appointments in four fields of science—biology, civil and environmental engineering, geography, and public health. We selected these four fields to ensure the diversity of scientific disciplines, as academic scientists may provide different perspectives on social issues. In the context of this article, public health and biology faculty can be expected to have more professional knowledge about the biological and public health dimensions of abortion and reproductive health, while engineers and geographers provide general insights as regular highly educated scientists. The SciOPS research team used probability sampling methods to randomly select R1 universities covering departments of the four fields in the United States. Supplementary Table A1 in Supplementary Appendix SA1 shows the number of institutions that were randomly selected by discipline.

For each sampled university, we collected the name and contact information of the tenured, tenure track, and nontenure track faculty from department websites. The research team discussed and determined the fields according to the major disciplines of the departments we sampled. The full sample frame for recruiting SciOPS panelists included contact information for around 12,000 scientists. This comprehensive sample represents the complete academic scientist population within the institutions and departments sampled. We sent a four-round recruitment invitation to this sample of scientists. Of these, 986 consented to become SciOPS panel members, with an AAPOR recruitment rate (RECR) of 8.1% (American Association for Public Opinion Research, 2023).

Next, we randomly selected 400 academic scientists from the 986 SciOPS panel members as the sample for this cross-sectional survey. We administered this survey in English using the online NubiS^®^ system, which is an accessible and versatile software system specifically designed to administer questionnaires with protection for the confidentiality of the survey respondents. Individuals were invited to participate in this survey *via* email invitation and two follow-up email reminders. An email prenotification message was sent on December 2, 2022, to notify sampled individuals that they would be receiving the questionnaire shortly. An email invitation (including unique ID, passwords, and hyperlink to the questionnaire) was sent on December 15, 2022, followed by two reminder emails on December 22, 2022, and January 3, 2023. To protect confidentiality and avoid multiple participation, each sampled individual had the own unique ID and password. The survey was closed on January 9, 2023, resulting in 149 responses, representing an AAPOR Individual Survey Completion Rate of 37.3%. The AAPOR Cumulative Response Rate, which accounts for nonresponse to initial panel recruitment in addition to this survey’s completion rate, was 3%^d^.^24^ We identified respondents whose percentage of answers to substantive survey items was below 60% and categorized them as partial responses, representing a 4% break off rate (6 of 149 cases).

The survey was designed, conducted, analyzed, and sponsored by the Center for Science, Technology, and Environmental Policy Studies at Arizona State University. There were 4 sections and 11 questions in the survey questionnaire, which is reproduced in Supplementary Appendix SA3. All study procedures were approved in advance by the Arizona State University Institutional Review Board (Study #00011868) and at the University of Illinois at Chicago (Protocol #2020–0470).

Table 1 reports descriptive statistics for respondents. Political leaning of the states at which scientist institutions are located are defined using the 2022 Cook Partisan Voting Index.^e^ We mapped in Figure 3 the state’s political leaning, with deeper blue indicating a greater leaning to the Democratic party and deeper red indicating a greater leaning to the Republican party.

**Table 1.** Descriptive Statistics

Construct	Variable	N	%
Gender	Female	81	54.4
	Male	68	45.6
Field	Biology	77	51.7
	Civil and environmental engineering	21	14.1
	Geography	15	10.1
	Public health	36	24.2
Rank	Full professor	57	38.3
	Associate professor	31	20.8
	Assistant professor	20	13.4
	Nontenure track researcher	41	27.5
Geographical Region^f^	Northeast	32	21.5
	Midwest	24	16.1
	South	56	37.6
	West	37	24.8
Political Leanings	Democratic	77	51.7
	Republican	72	48.3

### Analysis of sample composition and weighting[Fn fn1]

Because the respondents of this survey opted into the SciOPS panel and self-administered the survey voluntarily, there may be potential sources of nonresponse bias^26^. It is necessary to evaluate whether the composition of the final sample of respondents is representative of the sample frame for recruiting SciOPS panel members and also represents the 400 randomly selected individuals invited to participate in this survey. Two-sample t-tests using R software were conducted for these comparisons. Detailed results are reported in Supplementary Appendix SA2. Briefly, females and nontenure track researchers were significantly overrepresented, and assistant professors were underrepresented in the final sample of respondents for this survey (*p* value <0.005), relative to the composition of the recruitment sample frame. We did not observe any demographic differences across the final sample of respondents and the 400 panel members invited to participate.

To address the potential bias caused by these demographic discrepancies, the final sample of respondents was weighted by the inverse of selection probabilities and poststratified by gender and academic field to represent the recruitment sample frame as closely as possible. A conservative measure of sampling error for questions answered by the sample of respondents is ±8 percentage points. These weighting adjustments assume that respondents and nonrespondents with a given demographic characteristic will provide similar responses.^25^

## Results

### Survey results and comparison with other national surveys

**FIG. 1.**
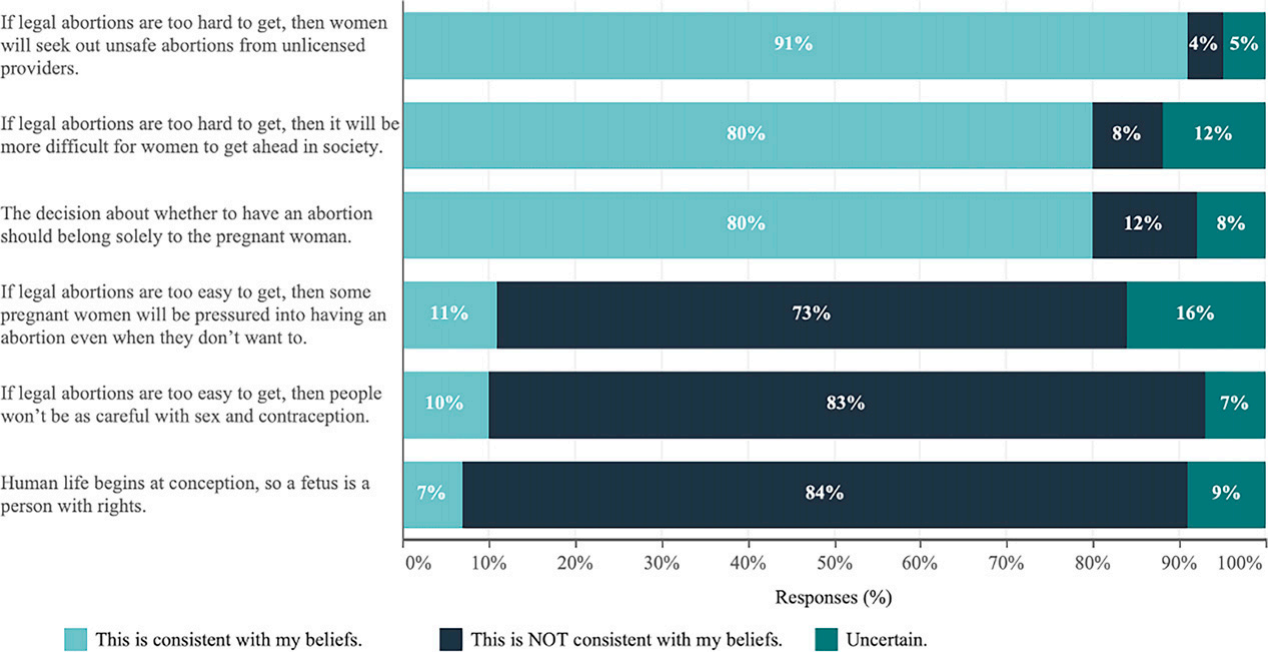
SciOPS survey responses to the question: A short list of statements regarding views on abortion is provided. For each statement, please indicate whether it is or is not consistent with your personal beliefs.

The main findings from the SciOPS survey indicate that most academic scientists (82%) considered abortion to be a very or an extremely important topic for their voting decision during the 2022 midterm election. Other topics that academic scientists felt were very or extremely important included the following: fair elections (81%), climate change (80%), and health care (71%). The SciOPS survey results also found that most academic scientists believe that if legal abortions are too hard to get, women will seek out unsafe abortions from unlicensed providers (91%) (see Fig. 1). A majority of scientists (80%) agreed with the statements “if legal abortions are too hard to get, it will be difficult for women to get ahead in society,” and “the decision to have an abortion belongs solely to the pregnant woman.” Also, a majority of academic scientists disagreed with the statement that “if legal abortions are too easy to get, then some pregnant women will be pressured into having an abortion even when they don’t want to” (73%) and “then people won’t be as careful with sex and contraception” (83%). Most academic scientists surveyed also disagreed that “a fetus is a person with rights” (84%) (see Fig. 1 for all previous statements).

**FIG. 2.**
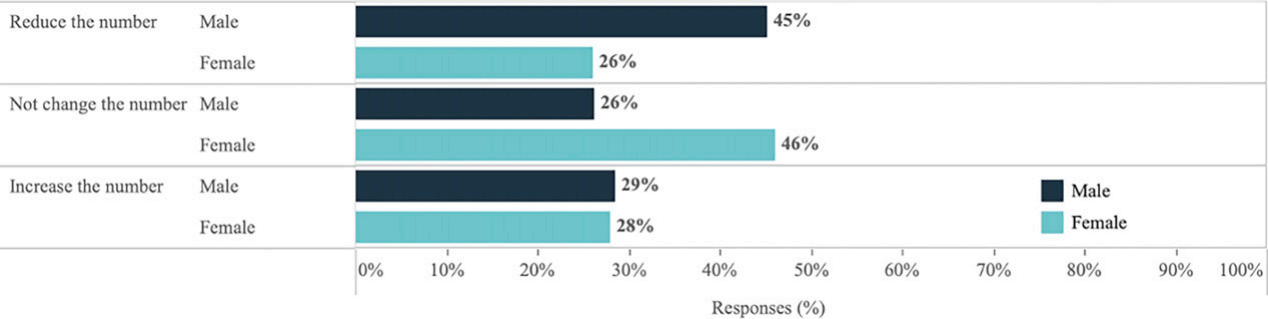
Gender differences in scientists’ opinions on the effect of “expanding sex education” on changing the number of abortions in the United States.

The SciOPS survey also found gender differences in academic scientists’ opinions regarding the effect of “expanding sex education” on changing the number of abortions in the United States (see Fig. 2). Almost half of all male academic scientists (45%) believed that expanding sex education would reduce the number of abortions in the United States, compared with only 26% of female academic scientists. On the contrary, 46% of female scientists indicated that expanding sex education would not change the number of abortions in the United States, compared with 26% of male scientists. These gender differences were significant (*p* = 0.04).

**FIG. 3.**
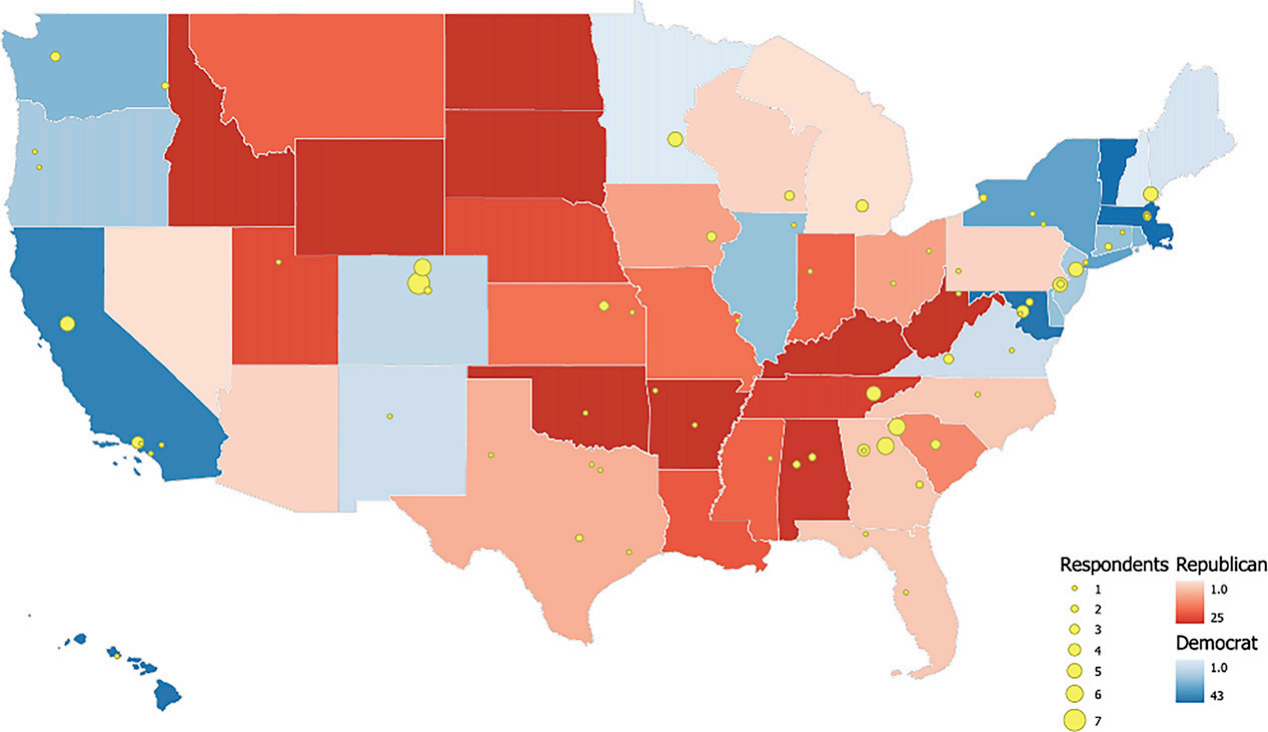
SciOPS survey respondents by universities and political view by states.^g^

The SciOPS survey also enabled us to examine political party differences. We focus on state policies and political leanings, however, rather than individual-level differences. Figure 3 shows a breakdown of SciOPS survey respondents by universities across the country and by their universities’ state political leaning. While multiple studies look at political party differences when it comes to the abortion debate, our analysis goes further to include a gendered analysis, which shows significant results across state political leanings. The survey asked how will the 2022 Supreme Court’s decision to overturn Roe v. Wade increase or decrease each of the following in your state? All results showed a significant difference by gender and state political leaning at *p* < 0.05 (see Table 2). For example, female respondents from Democratic states do not think access to low-cost or no-cost abortion services, to abortion services in general, and to reproductive health care in general will change at all. In contrast, women and men in Republican states[Fn fn1] believe that these services will decrease. In comparison, male respondents in Democratic states tend to have more mixed beliefs compared with their female counterparts. Understanding the nuanced interplay between state policies and individual beliefs shows the critical role of state-level political leanings in shaping the broader societal discourse surrounding contentious issues such as abortion.

**Table 2.** How Will the 2022 Supreme Court’s Decision to Overturn *Roe v. Wade* Increase or Decrease Each of the Following in Your State?

	Access to low-cost or no-cost abortion services		Access to abortion services in general		Likelihood of pregnancy-related death		Likelihood of pregnancy-related complications		Health inequities in general		Reproductive health care in general		Government interference in personal health care decisions	
	Dem	Rep	Dem	Rep	Dem	Rep	Dem	Rep	Dem	Rep	Dem	Rep	Dem	Rep
Male	Mixed	Decrease	Mixed	Decrease	Mixed	Increase	Mixed	Increase	Mixed	Increase	No change	Decrease	Mixed	Increase
Female	No change	Decrease	No change	Decrease	Increase	Increase	No change	Increase	No change	Increase	No change	Increase	Increase	Increase
Chi-square *p* value	0.017		0.030		0.049		0.011		0.048		0.024		<0.000	

Academic scientists were also asked about their workplace and whether the 2022 Supreme Court decision *Dobbs v. Jackson* has impacted the work environment within their university. Only 26% of the academic scientists reported that their university posted a public statement in response to the *Dobbs* decision, while 36% responded that their university did not post a public statement in response, and the remaining 38% did not know. Less than 10% of the scientists reported that their department provided resources (7%) or organized an information session for students (6%) in response to the *Dobbs* decision. Less than 5% reported that *Dobbs v. Jackson* was formally discussed in faculty meetings (4%) or that an information session was organized for the faculty (4%).

When asked about whom they spoke to on campus about the decision’s impact, 50% answered that they talked to other faculty or teachers in their department or school. There was a significant difference by gender (*p* = 0.04), with more female academic scientists (61%) having spoken to faculty or others in their own department or school, compared with 43% of male academic scientists. Almost one-third had talked to graduate students (31%), while 17% had talked to undergraduate students.

**FIG. 4.**
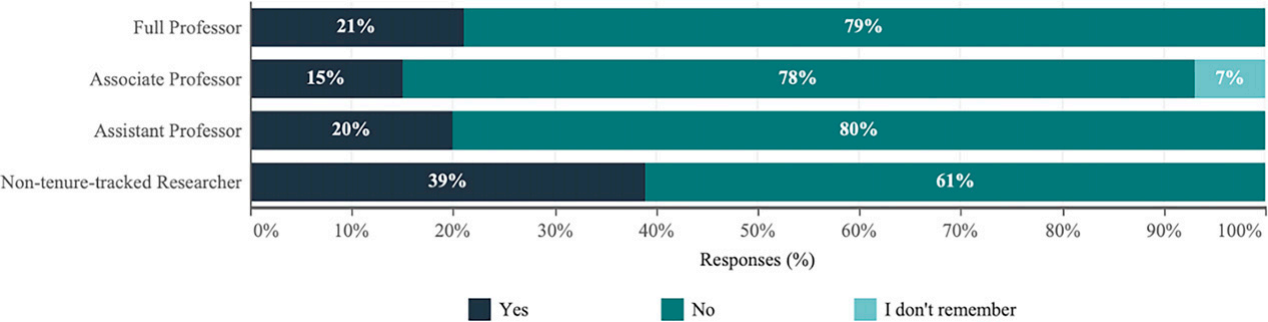
Comparison by academic rank for posting a comment on social media to respond to the 2022 Supreme Court decision.

When asked how they had personally responded to the *Dobbs* decision, the two most common actions taken were signing a petition (35%) and posting comments on social media (24%). A small proportion of academic scientists attended or organized gatherings or protests off campus (14%) or campaigned for a candidate in local (13%) or national elections (11%). There was a significant difference by academic rank regarding the posting of comments on social media in response to *Dobbs v. Jackson* (*p* = 0.033). The proportion of nontenure-track researchers who posted comments on their social media in response to the *Dobbs* decision was higher (39%) than other faculty (see Fig. 4).

## Discussion

Using a nationally representative survey sample, we present the opinions of academic scientists about the impacts of the 2022 Supreme Court *Dobbs v. Jackson* decision as well as relevant state and university policies. The SciOPS survey illustrates how abortion rights continue to be a key political topic with significant implications for reproductive health. The survey results highlight the views of academic scientists, who tend to have a greater understanding of important biological and public health elements of the abortion debate,^17,24^ which facilitates comparisons with the views of the general population.

**FIG. 5.**
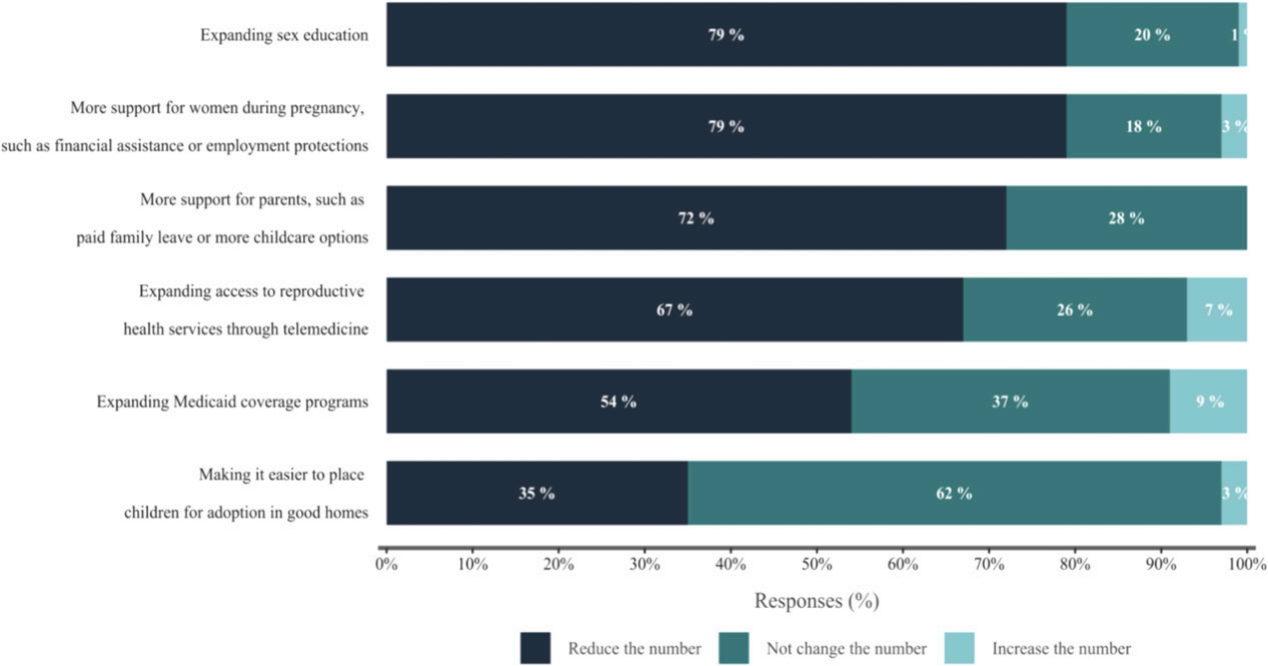
SciOPS survey responses to the question: How much, if at all, do you think each of the following would change the number of abortions in the United States?

The SciOPS findings can be directly compared with a PEW Research (2022) survey^11^ that interviewed a representative sample of the American public, which asked some of the same questions^h^. The SciOPS survey respondents were more likely to favor providing support for women as a means to reduce the number of abortions, in comparison with PEW (2022) survey respondents. For example, 79% of SciOPS respondents reported that increasing the support for women during pregnancy and expanding sex education are effective ways of reducing the number of abortions compared with around 60% of PEW respondents (see Table 3). In addition, more than half of SciOPS respondents think that the 2022 Supreme Court’s decision will decrease access to low-cost or no-cost abortion services (57%) and general abortion services (59%) (see Fig. 5).

**Table 3.** Direct Comparison of SciOPS and PEW Survey Results

Survey questions	SciOPS survey scientists	PEW survey general public
Please indicate whether it is or is not consistent with your personal beliefs.		
“Human life begins at conception, so a fetus is a person with rights”	84% state it is not consistent with my beliefs	42% state it is not consistent with my beliefs
“If legal abortions are too easy to get, then some pregnant women will be pressured into having an abortion even when they don’t want to”	73% state it is not consistent with my beliefs	42% state it is not consistent with my beliefs
How much, if at all, do you think each of the following would change the number of abortions in the United States?		
Support for women during pregnancy	79%	65%
Expanding sex education	79%	60%
More support for parents, such as paid family leave or more child care options	72%	58%

In addition, 82% of the surveyed academic scientists compared with 56% of the surveyed general public considered abortion to be very important in their midterm voting decisions. These differing views on key issues, including abortion rights, highlight a disparity between the opinions of the scientific community and those of the broader public. Greater dissemination and knowledge of these differences have the potential to influence voting decisions. While studies find that academic scientists should play a key role in policy discussions due to the nature of the subject,^4^ our study implies a lack of interaction as there is a disconnect between what experts and the general public know. Addressing this gap in understanding could be done by providing access to expert knowledge and views on politically relevant matters that have scientific implications. This could involve actively engaging and consulting with academic scientists, and disseminating their insights more widely, besides through repeated media channels.

Another key finding is that both the SciOPS and PEW (2022) surveys^11^ find gender differences in support for legal abortion and consideration of alternative strategies, such as sex education. More male academic scientists believe “expanding sex education” would reduce the number of abortions in the United States (see Fig. 3). While the PEW (2022) survey has somewhat muted gender findings due to broader demographic factors such as age and political views, there are still key gender differences. For example, in that survey, more men believe “passing stricter laws” would reduce the number of abortions in the United States compared with women.^i^ The gender comparisons from both surveys show that while there are clear policy disagreements between the scientific community and the general public, important gender differences remain among both academic scientists and the general public respondents. These gender differences exist as well by state political leanings (see Fig. 3), an area for further investigation.

Other key findings from the SciOPS survey indicate that, while most scientists (82%) agreed that abortion was a very or an extremely important consideration when making voting decisions in the 2022 midterm election, very few took personal action in response. The two most common actions taken after the 2022 Supreme Court’s decision were signing a petition (35%) and posting comments on social media (24%). Nontenure-track researchers were more likely to post comments on social media, possibly due to being younger on average and/or less fear of facing professional backlash, although the overall institutional conservatism of universities acculturates researchers to not be vocal or politically active.^23^ While the general public would benefit from the involvement of academic scientists in political discourse, the academics themselves can face both formal and informal institutional consequences for such actions.^27^ Universities could help to encourage academic scientists to engage appropriately in public discussions by limiting formal policies that could censor scientist commentary on current events.

While the survey sample was based on probability sampling, limitations include the relatively small final sample of academic scientists and that findings are limited to a relatively small set of academic fields, including biology, civil and environmental engineering, geography, and public health. Nonetheless, the findings remain representative of academic scientists within a set of academic disciplines sampled and aligned with many of the trends of similar nationally representative surveys.^13,14^

There remains significant interest in understanding changes to reproductive rights due to the 2022 Supreme Court decision and differing state and university policy choices. It is valuable to consider the input of the scientific community during national debates, such as on reproductive rights, as academic scientists are experts with insights and perspectives that may differ from the general public. Scientists have the expertise and specialized knowledge from respective fields to provide policy makers and voters with valuable information, and as such are seen as a credible source.^4^ In addition, as universities are generally not taking specific policy positions on such a sensitive topic, more of the burden to be outspoken falls on faculty, particularly female scientists. Both the lack of diversity in STEM fields^20^ and the personal impact this topic can have on female faculty put them in a distinct position, warranting attention and focus compared with their counterparts.

## Supplementary Material

Supplementary Appendix SA1

Supplementary Appendix SA2

Supplementary Appendix SA3

Supplementary Table SA1

## Notes

^a^
*Roe v. Wade*, 410 U.S. 113 (1973)

^b^
*Dobbs v. Jackson Women’s Health Organization*, 597 U.S. ___ (2022)

^c^
The SciOPS (Scientist Opinion Panel Survey) panel is a science communication platform designed to provide science expert opinions on timely and important topics related to science and the public. The SciOPS panel is recruited from randomly selected scientists and researchers employed at Research Intensive (R1) universities in the United States. Please find detailed information regarding SciOPS at: https://www.sci-ops.org/

^d^
The AAPOR Cumulative Response Rate is the product of the SciOPS panel recruitment rate (8.1%) multiplied by the Individual Survey Completion Rate for this survey (37.3%) (American Association for Public Opinion Research, 2023).

^e^
Data source: https://www.cookpolitical.com/cook-pvi/2022-partisan-voting-index/state-map-and-list

^f^
The geographic division is based on the criteria made by the U.S. Census Bureau. https://www.cdc.gov/nchs/hus/sources-definitions/geographic-region.htm

^g^
State political leaning gradient scale used to design the map is based on the Cook 2022 report. The yellow dots on the map illustrate the number of respondents per university per state.

^h^
SciOPS survey instrument includes many of the same survey questions from the PEW Research (2022) survey.

^i^

https://www.pewresearch.org/short-reads/2022/06/23/americans-differ-by-party-age-over-ways-to-reduce-the-number-of-abortions-in-the-u-s/

## Abbreviations Used

AAPOR: American Association for Public Opinion Research
US: United States
