# Supplementary material for: Abortion Rights: Perspectives of Academic Scientists in the United States
Source: Womens Health Rep (New Rochelle). 2024 Sep 4;5(1):602–12. doi: 10.1089/whr.2024.0041 (PMC11513566; doi:10.1089/whr.2024.0041)
Supplement: Supplementary Appendix SA1 [file whr.2024.0041_supplementaryappendix.pdf]

Appendix A. SciOPS panel recruitment

Table A. Number of randomly selected institutions for SciOPS panel recruitment sampling scientists

Fields	Number of randomly selected R1 institutions	Number of all R1 institutions
Biology	106	131
Civil and environmental engineering	46	131
Geography	46	131
Public health	61	61

Appendix B. Analysis of sample composition

We conducted two sets of analyses to examine similarities and differences in the composition of our final sample when compared with the initial recruitment panel and with the full sample invited to participate in this survey. Table B.1 shows t-test comparisons for demographic differences. Females were significantly over-represented in the final sample of respondents for this survey (p-value < 0.005), relative to the composition of the full sample frame for recruiting SciOPS panel members. There were no observed differences between respondents and the recruitment sample for most academic fields (i.e., Biology, Geography, and Public Health), except there was a borderline significant difference (p-value = 0.073) with engineers being slightly under-represented in the panelist sample. We also observed differences by academic ranking as assistant professors were under-represented in the final sample of respondents (p-value < 0.005) and Non-tenure track researchers are over-represented (p-value < 0.005). There are no observed differences between respondents and the initial recruitment sample by political leaning of the state within which their university was located.

Table B.1. T-test results for demographic differences between respondents and initial recruitment sample

**Construct**	**Variable**	**Recruitment sample (%)**	**Respondents (%)**	**Groups Differences (%)**	**P-value**
Gender	Female	37.1	54.4	17.3***	<0.005
Field	Biology	47.4	51.7	4.3	0.303
	Civil and Environmental Engineering	19.3	14.1	5.2	0.073
	Geography	6.2	10.1	3.9	0.123
	Public Health	27.1	24.2	2.9	0.412
Rank	Full Professor	40.9	38.3	2.7	0.506
	Associate Professor	22.9	20.8	2.1	0.536
	Assistant Professor	21.9	13.4	8.5***	0.003
	Non-tenure Track Researcher	14.2	27.5	13.3***	<0.005
Political leanings	Republican	53.1	48.3	0.04	0.253
Observation		12403	149		

To evaluate potential non-response bias, we compared the sample composition of the final sample of respondents with the 400 randomly selected sample for this survey. Sample t-tests were calculated to see whether there were statistically significant differences across each demographic measure between sample groups. The basic assumption behind this analysis was that scientists' demographic characteristics are relevant to their responses to other survey questions. If there is an observed demographic difference, nonresponse bias might be present. Table B.2 shows t-test results. We did not observe significant differences for any demographic characteristics and for political leanings across these two groups, although borderline significant differences were noted, with with females (p-value = 0.082) and non-tenure track researchers (p-value = 0.095) found to be slightly over-represented in the final sample of respondents.

Table B.2. T-test results for demographic differences between respondents and survey sample

Construct	Variable	Survey sample (%)	Respondents (%)	Groups Differences (%)	P-value
Gender	Female	46	54.4	8.4	0.082
Field	Biology	45	51.7	6.7	0.166
	Civil and Environmental Engineering	18	14.1	3.9	0.258
	Geography	10.2	10.1	0.2	0.95
	Public Health	26.8	24.2	2.6	0.534
Rank	Full Professor	39	38.3	0.7	0.874
	Associate Professor	22.5	20.8	1.7	0.667
	Assistant Professor	18	13.4	4.6	0.179
	Non-tenure Track Researcher	20.5	27.5	7	0.095
Political leanings	Republican	48.3	48.3	0	0.988
Observation		400	149		

Appendix C. Survey instrument

Section I: Your Vote in the 2022 General Election

Thank you for participating in this survey!
We would first like to ask about the topics that you cared about when you voted in the last general election in November 2022. If you did not vote, you will skip these questions after your response below.

(Section1Q1) Did you vote in the 2022 general election or not?

1= Yes, did vote
2=Not, did not vote
3= I prefer not to answer

[If YES in Section1Q1 then, Section1Q2, else, then Section2.]

(Section1Q2) How important were each of the following topics when you made your decision regarding which candidates to vote for in the 2022 midterm election?
[1=Not at all important, 2=Not very important, 3=Somewhat important, 4=Very important, 5=Extremely important]

a. Climate change
b. Inflation
c. Abortion
d. Healthcare
e. Fair elections
f. Immigration
g. Coronavirus
h. The war in Ukraine

Section II: Your Personal Views
In this section, we ask about your views on abortion and women's access to reproductive health care.

(Section2Q1) Following is a short list of statements regarding views on abortion. For each, please indicate whether it is or is not consistent with your personal beliefs.
[1 = This is NOT consistent with my beliefs. 2 = This is consistent with my beliefs. 3=Uncertain.]

a. The decision about whether to have an abortion should belong solely to the pregnant woman.
b. If legal abortions are too hard to get, then women will seek out unsafe abortions from unlicensed providers.
c. If legal abortions are too hard to get, then it will be more difficult for women to get ahead in society.

d. If legal abortions are too easy to get, then people won’t be as careful with sex and contraception.
e. If legal abortions are too easy to get, then some pregnant women will be pressured into having an abortion even when they don’t want to.
f. Human life begins at conception, so a fetus is a person with rights.

(Section2Q2) How much, if at all, do you think each of the following would change the number of abortions in the U.S.?
[1=Greatly reduce the number, 2=Slightly reduce the number, 3=Not change the number, 4=Slightly increase the number, 5=Greatly increase the number]

a. Expanding access to reproductive health services through telemedicine
b. Expanding Medicaid coverage programs
c. More support for parents, such as paid family leave or more childcare options
d. Expanding sex education
e. Making it easier to place children for adoption in good homes
f. More support for women during pregnancy, such as financial assistance or employment protections

Section III: State Policies
This section asks about your views on the possible effects of the 2022 Supreme Court's decision in *Dobbs v. Jackson Women’s Health Organization* on women's reproductive health outcomes and policies in your state.

(Section3Q1) How will the 2022 Supreme Court's decision to overturn *Roe v. Wade* increase or decrease each of the following in your state?
[1=Decrease substantially, 2=Decrease somewhat, 3=No change, 4=Increase somewhat, 5=Increase substantially]

a. Access to low cost or no cost abortion services
b. Access to abortion services in general
c. Likelihood of pregnancy related death
d. Likelihood of pregnancy-related complications
e. Health inequities in general
f. Reproductive health care in general
g. Government interference in personal healthcare decisions

(Section3Q2) Does your state currently have a law or laws that protect women’s right to abortion?
1=Yes
2=No
3=I don’t know

(Section3Q3) In your opinion, in the next two years will your state pass new abortion legislation that is more restrictive or less restrictive than the current law?

1=Will pass less restrictive abortion legislation.
2=Will pass more restrictive abortion legislation.
3=Uncertain.

Section IV: Your Workplace and You

Thank you so much for your participation. This last section asks about how the 2022 Supreme Court's decision in *Dobbs v. Jackson Women's Health Organization* has affected the work environment in your university.

(Section4Q1) Has your university posted a public statement in response to the 2022 Supreme Court's decision in *Dobbs v. Jackson Women's Health Organization*?

1=Yes - did post a public statement
2=No - did not post a public statement
3=I don't know

(Section4Q2) Have you talked to any of the following people on your campus about how the 2022 Supreme Court's decision in *Dobbs v. Jackson Women's Health Organization* affects universities?

[1=Yes, 2=No, 3=I don't remember]

a. Undergraduate students
b. Graduate students
c. Postdoctoral fellows or scholars
d. Faculty or teachers in your department or school
e. Faculty or teachers in other departments or schools
f. University staff
g. University administrators

(Section4Q3) As a response to the 2022 Supreme Court's decision in *Dobbs v. Jackson Women's Health Organization*, have you done any of the following?

[1=Yes, 2=No, 3=I don't remember]

a. Sent a letter to the editor of a local newspaper
b. Signed a petition
c. Posted a comment on social media
d. Attended an organized gathering or protest on campus
e. Attended an organized gathering or protest off campus
f. Campaigned for a candidate in the local election
g. Campaigned for a candidate in the national election
h. Other (please specify, if any) __________________

(Section4Q4) As a response to the 2022 Supreme Court's decision in *Dobbs v. Jackson Women's Health Organization*, has your department done any of the following?

[1=Yes, 2=No, 3=I don't know]

a. Formally discussed in faculty meetings
b. Provided a list of resources to students
c. Organized an information session for students
d. Organized an information session for staff or faculty
e. Other (please specify, if any) __________________
